# St. George's and Westminster Hospitals' Amalgamation

**Published:** 1913-07-19

**Authors:** 


### The Hospital

The Workers' Newspaper of Administrative Medicine and Institutional Life, Administration, National Insurance and Health.

No. 1412, Vol. LIV. SATURDAY, JULY 19, 1913. PRICE ONE PENNY.

### ST. GEORGE'S AND WESTMINSTER HOSPITALS' AMALGAMATION.

A meeting of the governors of St. George's Hospital on the 10th inst. unanimously passed the resolutions, which we published last week, approving the amalgamation with the Westminster Hospital. This is a most momentous decision and reflects credit upon Mr. West, the treasurer, who for some years has quietly but firmly worked away in support of a policy of administrative reform, and to secure the maximum of efficiency and benefit to St. George's Hospital and the interests of the patients for whose benefit it was established. The report of the proceedings at the meeting of governors, which we publish elsewhere, will set forth fully the facts, so far as they are available at the moment. It was elicited in the course of the discussion, that St. George's Hospital is not committed definitely to any particular site, though two available sites were mentioned, one at Wandsworth and one at Clapham. Mr. West expressed himself in favour of the Wandsworth site, but an old and most cautious and well-informed governor, Mr. Thorp, pointed out that the majority of the patients at present come from the Walham Green district, and that there are no means of communication at present which would give these patients easy access to the hospital if placed upon the Wandsworth site. Their only means of reaching it would be by walking quite a mile and a half across Wandsworth Bridge, which seems to point to the need for reconsideration, and the making of special arrangements for tramway and 'bus facilities, before the Wandsworth site can prove satisfactory.

On the general principle there can be but one opinion, that the amalgamation of St. George's and Westminster Hospitals is desirable in the interests of the patients, the adequate development of the resources of the two institutions in combination, and the full up-to-date efficiency of the medical school. It has taken a good many years to overcome the sentiment which animated a great many governors of St. George's Hospital in regard to the site and their attachment to the old but obsolete plan of the open Board. The first thing which had to be secured was the appointment of a House Committee to manage the affairs of the hospital, and one result of the abolition of the open Board has been to bring about the unanimous approval of the present scheme for amalgamation, which, if carefully organised and wisely carried out, is full of promise for the future.

It is gratifying to hear that the Duke of Westminster and his Board, the Charity Commissioners, and the medical staff and authorities of the Westminster Hospital have entered *con amore* into the amalgamation proposals, and that the combined hospitals will have invested funds amounting to upwards of £800,000 when the amalgamation is completed. It is further satisfactory to know arrangements can be made whereby an entirely new and up-to-date modern hospital, to contain at least 500 beds, can be erected and made ready for the reception of patients before the date on which the authorities will have to give up possession of the present hospital and school buildings. Something was said about the present site being good in the financial sense, because so many people of wealth and means became familiar with it as they entered and left Hyde Park. We have often examined this claim, with the result that we have reason to believe that financially it cannot be substantiated in fact. Either of the sites suggested will make the hospital serve the needs of a poor population of some half million of people—a fact which should bring much popularity and more money in support of the combined hospitals in the future than they have ever received in the past. Once they are amalgamated and a new modern hospital with at least 500 beds is erected, with the addition of a perfectly organised, well-planned medical school in close proximity, the increased usefulness and importance of such a hospital cannot fail to bring to it a hearty welcome and to secure for it the sympathy and support of many new friends. One very important point has yet to be cleared up. How is the money to be provided to defray the cost of building and equipping the new medical school and technical departments of the amalgamated hospital?

The present position of our voluntary hospital system is one of some doubt and no little difficulty. This fact affords an excellent opportunity for the managers of the amalgamated hospitals so to reorganise and settle their scheme of work, and the objects of the new hospital, as to secure for them the fullest usefulness and attraction to all classes of the community who may need hospital care. In defining the objects Sir William Osler's address at Oxford will no doubt be fully noted. At the present time the breadwinners are embraced by National Insurance, but within a short interval their wives and children are likely to be embraced too. What grounds will there then be to warrant the free gift of hospital treatment to insured persons? With the reform of the Poor Law, which is already overdue, what type of people would be left, under a system of National Insurance organised on a business basis, to claim free hospital care and justify the free gifts of the benevolent to pay for it? These considerations must force upon the governors of the amalgamated hospitals the necessity for providing a system of pay wards for the accommodation of various classes of the community, including insured persons. The amalgamated hospitals have a great opportunity for showing the way to a practical reorganisation of our present hospital system upon a new plan. This will preserve and strengthen the voluntary principle, whilst it provides much-needed hospital accommodation for all classes of the community who are willing to contribute towards the cost of their hospital treatment.

